# Nutrition risk among an ethnically diverse sample of community-dwelling older adults

**DOI:** 10.1017/S1368980018002902

**Published:** 2018-11-08

**Authors:** Patricia Sheean, Isabel C Farrar, Suela Sulo, Jamie Partridge, Linda Schiffer, Marian Fitzgibbon

**Affiliations:** 1 Marcella Niehoff School of Nursing, Loyola University Chicago, 2160 South First Avenue, Building 120, Room 4527, Maywood, IL 60153, USA; 2 Survey Research Laboratory, University of Illinois at Chicago, Chicago, IL, USA; 3 Abbott Nutrition Research & Development, Columbus, OH, USA; 4 Institute for Health Research and Policy, University of Illinois at Chicago, Chicago, IL, USA; 5 Department of Pediatrics, College of Medicine, University of Illinois at Chicago, Chicago, IL, USA

**Keywords:** Nutrition risk, Community nutrition, Older adults, Minority health, Urban environment

## Abstract

**Objective:**

To assess the prevalence of nutritional risk among an ethnically diverse group of urban community-dwelling older adults and to explore if risk varied by race/ethnicity.

**Design:**

Demographic characteristics, Katz’s activities of daily living and health-care resource utilization were ascertained cross-sectionally via telephone surveys with trained interviewers. Nutrition risk and nutrition symptomology were assessed via the abridged Patient Generated Subjective Global Assessment (abPG-SGA); scores of ≥6 points delineated ‘high’ nutrition risk. Descriptive statistics and logistic regression analyses were conducted.

**Setting:**

Urban.

**Participants:**

White, Black or Hispanic community-dwelling adults, ≥55 years of age, fluent in English or Spanish, residing in the city limits of Chicago, IL, USA.

**Results:**

A total of 1001 participants (37 % white, 37 % Black, 26 % Hispanic) were surveyed. On average, participants were 66·9 years old, predominantly female and overweight/obese. Twenty-six per cent (*n* 263) of participants were classified as ‘high’ nutrition risk with 24, 14 and 31 % endorsing decreased oral intake, weight loss and compromised functioning, respectively. Black respondents constituted the greatest proportion of those with high risk scores, yet Hispanic participants displayed the most concerning nutrition risk profiles. Younger age, female sex, Black or Hispanic race/ethnicity, emergency room visits, eating alone and taking three or more different prescribed or over-the-counter drugs daily were significantly associated with high risk scores (*P*<0·05).

**Conclusions:**

One in four older adults living in an urban community prone to health disparities was classified as ‘high’ nutrition risk. Targeted interventions to promote healthy ageing are needed, especially for overweight/obese and minority community members.

Compromises in nutritional status can be a challenge to healthy ageing among community-dwelling older adults^(^ ^1^ ^)^. In contrast to the numerous reports from acute care settings, the prevalence of nutritional risk in the community setting is less well established. This is somewhat surprising, considering the growth of the ageing population, the resultant mounting chronic disease burden and the simple fact that most individuals live independently (i.e. not in an acute or long-term care setting). Nevertheless, from a practical standpoint, assessing nutrition risk in the community setting is complex. First, there are a multitude of nutrition screening tools available. Therefore selecting the most appropriate is not necessarily straightforward, as each captures different risk profiles of target populations and has varying reliability^(^ ^2^ ^,^ ^3^ ^)^. Second, from an economic standpoint, it is a challenge to provide dedicated staff time to administer a nutrition screening tool, especially in a busy primary care office or geriatrics practice. Finally, in the event the nutrition screen identifies problems that could require immediate intervention or follow-up, access to appropriate resources in the community setting may be a challenge for patients and/or clinical staff (e.g. registered dietitians, affordable foods, supplements, clinical testing, etc.). Regardless, nutrition screening is considered a simple, yet critical first step in identifying if there are sufficient risk factors to warrant a more in-depth nutrition assessment, which includes physical examination and functional measures to substantiate a diagnosis of malnutrition^(^ ^4^ ^)^.

Several investigators have examined nutrition risk within the community setting using different approaches; however, most of the investigations have focused on free-living adults outside the USA. For example, Kvamme *et al*.^(^ ^5^ ^)^ evaluated the associations between Zn status and the risk of malnutrition in a cross-sectional sample of 1521 persons from Norway between the ages of 65 and 87 years. Using the Malnutrition Universal Screening Tool (MUST) and requiring participants to travel to the study centre for evaluation, 8 % (*n* 122) of participants were classified ‘at nutritional risk’. Of these, 10 % (*n* 81) were women and 5·5 % (*n* 41) were men. Westergren *et al*.^(^ ^6^ ^)^ evaluated the frequency of malnutrition risk and falling in 465 Swedish persons (age range 73–90 years). Data were collected during preventive home visits and interviews were conducted by two trained visiting research nurses. These authors reported that 35 % of these individuals were at moderate nutritional risk and 30 % were at high nutritional risk after administering the Seniors in the Community: Risk Evaluation for Eating and Nutrition Questionnaire (SCREEN II). Further, Winter *et al*.^(^ ^7^ ^)^ reported that after the administration of the Mini Nutrition Assessment – Short Form (MNA-SF) in a general health clinic for community participants attending the ‘75 plus’ health clinic in Australia, 16 % (*n* 36) were deemed ‘at nutritional risk’. Although mean BMI was lower in those at risk, 34 % of those in the at-risk group were overweight or obese (BMI≥25·0 kg/m^2^). These studies highlight the variability among nutrition screening tools, the resources needed to gather these data for individuals living in the home setting and the lack of diversity of populations sampled. These factors pose limitations to applying and/or generalizing these findings to more diverse populations of community-dwelling older adults, especially within the USA.

Therefore, the purpose of the present telephone-based study was to assess the nutrition risks of an ethnically diverse group of community-dwelling older adults and to explore if risk varied by race/ethnicity. Unlike previous studies, we sought to obtain these data using resource-efficient methodologies that did not warrant in-person administration, physical examination and/or participant travel to a central location. We hypothesized that nutrition risk would be greatest among Black respondents compared with respondents of other race/ethnicities due to greater overall chronic disease burden^(^ ^8^ ^)^.

## Methods

The current cross-sectional study was conducted in collaboration with the Survey Research Laboratory (SRL) at the University of Illinois at Chicago. Data were collected by the Interviewing Service of America (ISA). The study was conducted specifically in Chicago, IL, USA; a homogeneous urban environment rich in ethnic variability and health disparities^(^ ^9^ ^)^.

### Interviewer training

Prior to participant enrolment, twenty-nine ISA interviewers and supervisors were trained by the SRL project coordinator. The training included a general orientation to the design and purpose of the study, instructions on gaining cooperation of the respondent, a question-by-question review of the survey instrument (with instructions on how to record answers and how to probe) and practice interviews. All field staff were supplied with an interviewer training manual covering all aspects of the data collection procedures. In addition, all interviewers attended an in-depth debriefing before and during data collection, which included additional review of the study objectives/background, practice interviews and constructive feedback.

### Field procedures

Interviews were conducted over several months by English- and Spanish-speaking, trained ISA interviewers. Four sample frames were included in the study sample: two listed sample frames (cell and landline) and two random-digit-dial sample frames (cell and landline). The random-digit-dial sample included unlisted numbers. The two listed phone frames were targeted listed samples; targeted to attain completed interviews with people in the needed age range and racial/ethnic groups. The two random-digit-dial frames included a randomly selected sample of cellular and landline phone numbers in the City of Chicago. Cellular and landline random-digit-dial phone numbers were included to give everyone (or, all those who have any type of phone associated with the City of Chicago) some probability of being included in the sample. This methodology increases population coverage over a design that includes only the listed sample or only the random-digit-dial sample. Calls to potential participants were prioritized to weekday evenings and weekends to increase the probability of successful contact with respondents. Up to six call attempts were made before finalizing a case as a ‘non-contact’.

### Participant eligibility and inclusion

Initial eligibility was established once the participant affirmed the following criteria: (i) 55 years of age or greater; (ii) self-reported White, Black or Hispanic; (iii) able to speak and understand English or Spanish; and (iv) currently living in the city limits of Chicago, IL, USA. Participants were included if they were willing to complete the remainder of the telephone survey or if they possessed a cellular phone with a Chicago area code but resided outside the geographic city limits.

### Questionnaire administration

Respondents were informed that the interview would require approximately 20 min to complete and incentives of $US 10 would be provided, unless the respondent declined to provide his/her mailing address. In addition to demographic items, respondents were asked about whom they resided with as well as access to and type of health-care insurance.

#### Nutritional risk assessment

To assess nutritional risk, interviewers administered the abridged Patient Generated Subjective Global Assessment (abPG-SGA), a screening tool typically used in the oncology setting^(^ ^10^ ^)^. However, unlike the MNA^(^ ^11^ ^)^, the abPG-SGA tool foregoes the physical exam as well as the disease condition and metabolic considerations of the conventional PG-SGA^(^ ^12^ ^)^. The abPG-SGA is unique in that it affords the opportunity to capture specific nutrition impact symptoms (e.g. anorexia, constipation, dry mouth, etc.). These symptoms are not captured in screening tools conventionally employed in community populations, such as the MUST^(^ ^5^ ^)^, DETERMINE^(^ ^13^ ^)^ or Short Nutritional Assessment Questionnaire 65+ (SNAQ65+)^(^ ^14^ ^)^, yet they are clinical important, relevant and easily administered over the phone. Specific symptoms, including weight loss, changes in oral intake and global decreases in physical functioning, as well as other responses are assigned numeric values, where higher scores reflect greater nutrition risk. Participants can complete the abPG-SGA in a minimum of eight or a maximum of twenty-three queries, depending upon current symptomology. Scores of ≥6 on the abPG-SGA are highly correlated with patients classified as malnourished when the full versions of the PG-SGA (97 % sensitivity, 86 % specificity, area under the curve=0·967) and the Malnutrition Screening Tool (81 % sensitivity, 72 % specificity, area under the curve=0·823) are employed^(^ ^10^ ^)^. In addition, we asked if participants ‘ate alone most of the time’ as a proxy of social isolation.

#### Functional assessment

Respondents were asked about potential functional impairments. Using the framing of Katz, participants were asked to rate themselves as dependent (i.e. needing supervision, direction, personal assistance or total care) or independent (i.e. no supervision, direction or personal assistance) in performing six activities of daily living^(^ ^15^ ^)^. Scores range from 0 to 6; higher scores indicate participant independence.

#### Health-care utilization

To explore potential associations between nutrition risk factors and health-care resources, respondents were asked a sequence of questions pertaining to health-care services received in the last 6 months. Participants were asked to respond to inquiries regarding: visits to the emergency room (frequency and medical reason), hospital, skilled nursing and/or inpatient rehabilitation facility admissions (frequency and admitting diagnosis), and the receipt of home care services (yes/no and if yes, rationale for services). In an attempt to get at the concept of polypharmacy, we also asked if participants ‘took three or more different prescribed or over-the-counter drugs per day’. This wording is supported by the DETERMINE tool^(^ ^13^ ^)^.

### Data processing and statistical analyses

Open-ended text data were translated from Spanish to English and all data were back-coded, which involved reviewing the all open-ended responses to determine if any could be coded into pre-existing response categories. Sample weights were applied to match the American Community Survey data based on 5-year estimates from 2011–2015 for age (55–64 and 65–75 years), gender (male or female) and by overall distribution of race/ethnicity (White, Black and Hispanic) in the City of Chicago^(^ ^16^ ^)^. Descriptive statistics were completed to: (i) examine participant characteristics and nutrition risk scores stratified by race/ethnicity; and (ii) assess the prevalence of nutrition risk symptoms stratified by race/ethnicity Additionally, we conducted some exploratory analyses to see if nutrition risk differed by sex and if obesity (defined as BMI≥30·0 kg/m^2^) was associated with specific nutrition risk factors among those deemed at high risk. Two-sample *t* tests were used for continuous variables and Pearson and *χ* ^2^ tests were conducted for categorical variables. Logistic regression analyses were conducted to determine the characteristics that independently predicted nutrition risk, taking account of significant covariates in the bivariate analyses and multicollinearity. All statistical analyses were conducted using the statistical software package SAS version 9.4 and a *P* value of 0·05 was used to denote statistical significance.

## Results

Of the 64 445 relevant phone numbers within the ISA database, the majority were classified as ‘no longer working’ (42 %, *n* 27 022), ‘no answer’ (27 %, *n* 17 427), ‘answering machine/voicemail’ (19 %, *n* 11 922) or ‘now living outside the City of Chicago’ (2 %, *n* 1010). Of the 7064 remaining numbers, 20 % (*n* 1430) were ‘ineligible’, 48 % (*n* 3402) were ‘not available/unable to screen’, 15 % (*n* 1081) ‘refused either before or after screening’ and 2 % (*n* 150) were screened but unable to interview. Two sets of response rates were calculated for the study. Using a ratio of completed interviews (*n* 1001) to the sum of cases known to be eligible (*n* 1166) and estimates of the number of eligible cases among the unknown eligible (*n* 11 783), the conservative resulting response rate was 7·7 %. However, if we included only the individuals who answered and cooperated with the screening process, our response rate was 85·8 % (*n* 1001/1166). The average number of call attempts per participant was 2·1. Interviews averaged 12·3 min in length and were conducted by ISA, in English or Spanish. Of the 1001 respondents who completed the interview, 845 (84·4 %) agreed to provide their mailing address to receive the incentive.

In general, the average study participant was 66·9 (sd 6·4) years of age, predominantly female (69 %), classified as overweight using BMI (29·5 (sd 6·7) kg/m^2^), educated (24 %, *n* 240 completed some college; 35 %, *n* 355 possessed a college and/or graduate degree), and largely independent with regard to activities of daily living (91 % reported autonomy in all six activities). For all participants, 17 % (*n* 172) reported visits to the emergency room, 13 % (*n* 128) were admitted to a hospital and 10 % (*n* 104) required home care services within the last 6 months. Further, 48 % (*n* 481) reported eating alone most of the time and nearly 56 % (*n* 559) reported taking three or more different prescribed or over-the-counter drugs daily.

Table 1 displays the characteristics of the study participants stratified by nutrition risk scores, revealing that 26 % (*n* 263) of participants were classified as high risk (≥6 points). These high-risk individuals were more likely to be female (*P*=0·01), minority (*P*<0·0001), less educated (*P*<0·0001) and obese (*P*=0·01). Additionally, participants classified as high risk were less independent in their activities of daily living (all *P*<0·05), reported significantly higher health-care utilization, more frequently ate alone (*P*<0·0001) and more frequently took three or more different prescribed or over-the-counter drugs daily (*P*<0·0001). Specifically, the prevalence of nutrition risk for White, Black and Hispanic participants was 16 % (*n* 60/373), 34 % (*n* 124/369) and 31 % (*n* 79/259), respectively. In a separate subgroup analyses of participants deemed at high nutrition risk, we examined if BMI was associated with any of the nutrition risk variables listed in Table 1. Although more females were classified as obese (*n* 93/251, *P*=0·02), BMI was not associated with nutrition risk. BMI was significantly associated with taking three or more different prescribed or over-the-counter drugs daily (*P*=0·002).

**Table 1:** Characteristics of urban community-dwelling older adults at low and high nutritional risk†, Chicago, IL, USA, August–October 2017

	Low nutrition risk (<6 points) (*N* 738)			High nutrition risk (≥6 points) (*N* 263)			
Variable	*N*	Mean or *n*	sd or %	*N*	Mean or *n*	sd or %	*P* ‡
Age (years)	697	67·2	6·4	255	66·3	6·3	0·06
Sex	738			263			0·01
Male		242	33		65	25	
Female		496	67		198	75	
Race/ethnicity	738			263			<0·0001
White		313	42		60	23	
Black		245	33		124	47	
Hispanic		180	24		79	30	
Education	676			234			<0·0001
Not HS graduate		100	15		51	22	
HS graduate or GED		121	18		43	18	
Some college		162	24		78	33	
College graduate		293	43		62	27	
BMI (kg/m^2^)	687	29·1	6·3	251	30·6	7·6	0·001
BMI category	687			251			0·01
Underweight (<18·5 kg/m^2^)		3	<1		4	2	
Normal weight (18·5–24·9 kg/m^2^)		192	28		52	21	
Overweight (25·0–29·9 kg/m^2^)		247	36		81	32	
Obese (≥30·0 kg/m^2^)		245	36		114	45	
Independence with ADL							
Bathing	736	718	98	262	246	94	0·005
Dressing	737	723	98	263	250	95	0·009
Toileting	737	727	99	263	256	97	0·17
Transferring	737	721	98	263	244	93	0·0001
Continence	730	707	97	260	235	90	<0·0001
Feeding	736	730	99	263	256	97	0·049
All 6 ADL	728	683	94	259	219	85	<0·0001
Health-care utilization							
ER visit	736	96	13	262	76	29	<0·0001
Hospital admission	735	81	11	263	47	18	0·004
SNF admission	735	11	2	263	6	2	0·41
Home care services	737	61	8	262	43	16	0·0002
Eat alone most of the time	736	317	43	261	164	63	<0·0001
Three or more different prescribed or OTC drugs per day	735	372	51	262	187	71	<0·0001

HS, high school; GED, General Equivalency Diploma; ADL, activities of daily living; ER, emergency room; SNF, skilled nursing facility; OTC, over-the-counter.

† Nutritional risk is defined using abridged Patient Generated Subjective Global Assessment (abPG-SGA) score: <6 points=low risk; ≥6 points=high risk.

‡ Tests for differences between low- and high-risk groups: *t* tests, with pooled variance for age and BMI, test for row mean score difference for ordinal variables (education and BMI categories); Fisher’s exact test for feeding, toileting and SNF admission; and *χ* ^2^ tests for other categorical variables. Responses of ‘don’t know’ or ‘refused’ are treated as missing and excluded from the denominator.

Table 2 presents the results of the logistic regression analyses conducted to predict nutrition risk. Younger age, female sex, minority race/ethnicity, emergency room visits, eating alone and taking three or more different prescribed or over-the-counter drugs daily were significantly associated with high nutrition risk, when adjusted for all other predictors included in the model. We tested the covariates for multicollinearity in the logistic regression model by examining the covariance matrix as well as the variance inflation factor and tolerance diagnostics. All correlation coefficients were <0·5, the variance inflation factor was <1·7 for all covariates and the tolerance was >0·6 for all covariates; thus, multicollinearity was not a significant concern for this model. Education did not appear to be significantly associated with nutrition risk overall (*P*=0·09), but participants with some college appeared to be at higher risk than college graduates (*P*=0·02).

**Table 2:** Logistic regression analyses for predicting nutritional risk among urban community-dwelling older adults†, Chicago, IL, USA, August–October 2017

	OR	95 % CI	*b*	*P*
Intercept		–	−1·507	0·16
Age, year	0·97	0·94, 1·00	−0·034	0·02
Female (ref.=male)	1·69	1·13, 2·51	0·522	0·01
Race (ref.=White)				0·0004
Black	2·41	1·55, 3·73	0·878	<0·0001
Hispanic	1·85	1·09, 3·14	0·616	0·02
Education (ref.=college graduate)				0·09
Not HS graduate	1·68	0·94, 3·01	0·521	0·08
HS graduate/GED	1·17	0·69, 1·98	0·155	0·57
Some college	1·69	1·07, 2·66	0·523	0·02
BMI, kg/m^2^	1·01	0·99, 1·04	0·011	0·41
Dependent for ≥1 ADL (ref.=independent for all 6 ADL)	1·36	0·72, 2·59	0·308	0·35
ER visit (ref.=none)	2·50	1·56, 4·00	0·915	0·0001
Hospital admission (ref.=none)	1·10	0·63, 1·90	0·091	0·74
Home care services (ref.=none)	1·22	0·69, 2·14	0·196	0·50
Eat alone most of the time (ref.=no)	2·36	1·66, 3·36	0·858	<0·0001
Take three or more different prescribed or OTC drugs per day (ref.=no)	2·33	1·59, 3·42	0·845	<0·0001

ref., reference category; HS, high school; GED, General Equivalency Diploma; ADL, activities of daily living; ER, emergency room; OTC, over-the-counter.

† From a logistic regression model predicting the risk of an abridged Patient Generated Subjective Global Assessment (abPG-SGA) score of ≥6 points (high nutrition risk). Some observations were excluded from the model due to missing data for covariates: *N* 810 (210 were high risk).

Table 3 examines specific components of the abPG-SGA by race/ethnicity and sex. Compared with White participants, Black participants reported eating less than usual (*P*<0·001) and experienced significantly more nutrition symptomology related to decreased appetite (*P*<0·001), constipation (*P*<0·001), taste changes (*P*<0·001), bothersome smells (*P*<0·001), early satiety (*P*<0·05) and pain (*P*<0·05). Compared with White respondents, Hispanic respondents reported significantly more nutrition symptomology related to decreased appetite (*P*<0·05), constipation (*P*<0·001), dry mouth (*P*<0·01), taste changes (*P*<0·001), bothersome smells (*P*<0·001), early satiety (*P*<0·01) and fatigue (*P*<0·01). Of the fifty-nine Hispanic participants who reported ‘less than usual intake’, they were consuming significantly little solid food (*P*<0·001), liquids (*P*<0·05), only nutrition supplements (*P*<0·01) and very little of anything (*P*<0·001), compared with the sixty-three White participants who reported ‘less than usual intake’. Black and Hispanic respondents reported significantly decreased physical functioning (‘not feeling up to most things’, *P*<0·05; ‘able to do little activity/bedridden’, *P*<0·001) compared with White respondents. When these nutrition variables were stratified by sex (male *v*. female), females reported a higher frequency of ‘no appetite’ (*P*=0·02) and ‘smells bothersome’ (*P*=0·003) compared with males.

**Table 3:** Prevalence of nutritional risk symptoms stratified by race/ethnicity and sex, using the components of the abridged Patient Generated Subjective Global Assessment (abPG-SGA), in urban community-dwelling older adults, Chicago, IL, USA, August–October 2017

		White (*N* 373)		Black (*N* 369)		Hispanic (*N* 259)			Male (*N* 307)		Female (*N* 694)		
Variable	*N*	*n*	%	*n*	%	*n*	%	*P* †	*n*	%	*n*	%	*P* ‡
During the past two weeks my weight has:	962							0·53					0·26
Decreased		44	12	52	15	36	15		44	15	88	13	
Not changed		293	81	270	76	189	77		226	75	526	79	
Increased		24	7	32	9	22	9		30	10	48	7	
Food intake§	995							<0·0001					0·10
Less than usual		63	17	113***	31	59	23		62	20	173	25	
Unchanged		272	73	220	60	177	69		211	69	458	66	
More than usual		37	10	32	9	22	9		33	11	58	8	
If less than usual intake, now consuming:	235	(*N* 63)		(*N* 113)		(*N* 59)			(*N* 62)		(*N* 173)		
Normal food	233	57	93	104	92	57	97	0·53	59	95	159	93	0·76
Little solid food	235	16	25	55**	49	39***	66	<0·0001	31	50	79	46	0·56
Only liquids	235	2	3	3	3	10*	17	0·003	6	10	9	5	0·23
Only nutrition supplements	234	3	5	13	12	13**	22	0·02	5	8	24	14	0·23
Very little of anything	228	11	18	34	31	33***	57	<0·0001	22	35	56	34	0·80
Tube or vein feedings	235	1	2	0	0	0	0	–	1	2	0	0	–
Symptoms													
No problems eating	1000	361	97	353	96	249	96	0·82	298	97	665	96	0·23
No appetite	998	57	15	101***	27	56*	22	0·0004	52	17	162	23	0·02
Nausea	1000	16	4	24	7	22	8	0·10	17	6	45	6	0·56
Vomiting	1001	5	1	6	2	10	4	0·08	6	2	15	2	0·83
Constipation	999	21	6	49***	13	61***	24	<0·0001	31	10	100	14	0·06
Diarrhoea	1000	31	8	32	9	20	8	0·91	24	8	59	9	0·71
Mouth sores	1001	12	3	7	2	4	2	0·33	10	3	13	2	0·18
Dry mouth	1000	35	9	36	10	47**	18	0·001	31	10	87	13	0·27
Things taste funny	997	13	4	50***	14	46***	18	<0·0001	36	12	73	11	0·58
Smells bothersome	994	18	5	47***	13	33***	13	0·0005	17	6	81	12	0·003
Problems swallowing	1001	10	3	12	3	9	3	0·83	10	3	21	3	0·85
Early satiety	991	102	28	130*	36	102**	39	0·008	92	30	242	35	0·13
Pain	998	17	5	34*	9	11	4	0·01	16	5	46	7	0·38
Fatigue	999	45	12	62	17	56**	22	0·007	48	16	115	17	0·70
Other problems	1000	12	3	14	4	7	3	0·75	9	3	24	3	0·67
Activities and function	993			***		**		0·0002					0·74
Normal without limitations		277	75	230	63	168	65		210	69	465	68	
Not my normal self		78	21	87	24	54	21		65	21	154	22	
Not feeling up to most things		12	3	25	7	17	7		17	6	37	5	
Able to do little activity/bedridden		3	1	24	7	18	7		13	4	32	5	

*P<0·05, **P<0·01 and ***P<0·001 are used to denote differences of Black and Hispanic participants where White participants are the referent population. All other *P* values reflect difference across all groups. Responses of ‘don’t know’ or ‘refused’ are treated as missing and excluded from the denominator.

† From logistic regression analyses (multinomial for weight change and ordinal for activities and function).

‡ From tests for row mean score differences for activities and function; Fisher’s exact test for variables with any expected cell values <5 and *χ* ^2^ tests for other variables.

§ For logistic regression and *χ* ^2^ analyses, ‘unchanged’ and ‘more than usual’ were combined and compared with ‘less than usual’.

## Discussion

In the present study which relied upon self-reported data, we found that 26 % of individuals who responded to our telephone survey had nutrition risk scores that would likely correlate with malnutrition classification^(^ ^10^ ^)^. Nutrition risk was higher among minority compared with non-minority participants, as evidenced by the significantly higher frequency of nutrition symptomology and decreased global physical functioning. Consistent with our hypothesis, Black respondents displayed a higher nutrition risk profiles compared with persons of other race/ethnicity, making an important contribution to the literature since previous studies have included predominantly non-minority participants. Similar to other studies, we also found that females were more likely to be at nutrition risk overall compared with men^(^ ^17^ ^,^ ^18^ ^)^. Because our sampling strategies only accounted for race/ethnicity and not sex, we cannot say if this reflects a true difference in nutrition risk or if females were more likely to respond to our survey requests. When we analysed the individual nutrition risk components by sex (Table 3), the nutrition risk profiles for females were comparable to male participants, supporting that this may be an artifact of higher study participation among females.

It is difficult to directly compare our prevalence estimates with others since different nutrition assessment tools were utilized and because our participants were not recruited from a hospital or clinic. However, our findings are similar to those for individuals living in a rural setting^(^ ^11^ ^)^, higher than those reported in older participants living at home^(^ ^19^ ^,^ ^20^ ^)^ and consistent with those of older hospitalized patients^(^ ^21^ ^–^ ^23^ ^)^. Together, these findings raise concerns that the nutrition issues of older adults residing in an urban, community setting are not well recognized. Older individuals possess a multitude of nutrition risk factors due to the physiological changes of ageing, including but not limited to: sensory losses (e.g. taste, smell, sight); alterations in gastrointestinal function (e.g. dysphagia, xerostomia, achlorhydria, delayed gastric emptying); adverse body composition changes (i.e. loss of lean mass, strength and function); decreased cognition; and the side-effects of medications often used to treat chronic diseases^(^ ^24^ ^)^. Considering the recent US Census Bureau report that the number of older individuals (≥65 years of age) residing in the USA grew by 40 % from 2000 to 2016^(^ ^25^ ^)^, our findings have significant public health implications. The Institute of Medicine identifies nutrition and the coordination of nutrition services in the community setting as integral components for promoting healthy ageing among older adults^(^ ^26^ ^)^.

The most recent consensus report by the Global Leadership Initiative on Malnutrition recommends the use of phenotypic and aetiological criteria be applied to diagnosis malnutrition^(^ ^27^ ^)^. The aetiology-based diagnosis classification includes malnutrition related to chronic disease with inflammation, chronic disease with minimal or no perceived inflammation, malnutrition related to acute disease or injury with severe inflammation or malnutrition related to starvation. Although limited by our methodologies, we speculate chronic-disease related malnutrition would be the most prevalent among our participants with high nutrition risk scores. Previous work supports that polypharmacy is associated with greater nutrition risk^(^ ^28^ ^–^ ^30^ ^)^. The majority of our participants (56 %) reported taking three or more different prescribed or over-the-counter drugs daily, lending support to the use of this crude metric as a proxy measure of co-morbid conditions and underlying chronic disease burden. In disease-related malnutrition, inflammation leads to a milieu of cytokine responses^(^ ^31^ ^)^, which adversely impact metabolism, appetite, dietary intake and body composition. Interestingly, we found that symptoms of ‘decreased weight’, ‘no appetite’ and ‘food intake less than usual’ were highly prevalent among participants (Table 3), which are clinical features that conventionally support a malnutrition diagnosis^(^ ^27^ ^)^. Additionally, 32 % (*n* 318) of participants reported limitations in their global performance status, which was further supported by an overall decrease in independence in activities of daily living among those with high nutrition risk scores. Given the high prevalence of obesity in our sample (36 %) and the inability of BMI to discriminate those who were high risk, these data are particularly worrisome for sarcopenic obesity; an occult condition of low lean mass and compromised function in the setting of obesity (BMI≥30·0 kg/m^2^)^(^ ^32^ ^)^. Using nationally representative data and dual-energy X-ray absorptiometry to quantify lean mass (kilograms), 24 % of older (≥60 years of age) adults residing in the community setting had sarcopenic obesity^(^ ^33^ ^)^. While nutrition screening would seem a logical first step to identifying individuals at risk, there tends to be a common perception that malnutrition is a problem restricted to the acute care setting and underweight patient populations^(^ ^34^ ^–^ ^36^ ^)^. Therefore, it is essential to raise awareness regarding the importance of identifying nutrition risks in both underweight and overweight adults, and further to reducing barriers to intervening through comprehensive nutrition-focused programmes implemented in community space or outpatient clinics. The Global Leadership Initiative on Malnutrition advocates assessing nutrition risks in common arenas (e.g. hospitals and nursing homes), as well as other health-care points of care^(^ ^37^ ^)^. Our data suggest the emergency room may be one unconventional venue to initiate screening and nutrition interventions in older individuals at high nutrition risk. Additionally, routine nutrition intervention and follow-up after hospitalization for high-risk individuals (i.e. critical illness, prolonged length of stay, etc.) or in those diagnosed with malnutrition seems paramount.

A recent systematic review by Hamirudin *et al*.^(^ ^38^ ^)^ highlights outcomes related to nutrition screening and nutrition interventions in older community-living adults. Over a span of 20 years, eleven nutrition interventions were identified and reviewed^(^ ^28^ ^,^ ^29^ ^,^ ^39^ ^–^ ^47^ ^)^; the prevalence of malnutrition was approximately 35 %. Nutrition interventions in these studies included access to or the provision of: healthy eating flyers^(^ ^28^ ^,^ ^29^ ^,^ ^42^ ^,^ ^46^ ^)^, nutrition counselling (in person or telephone)^(^ ^1^ ^,^ ^39^ ^,^ ^41^ ^,^ ^43^ ^–^ ^45^ ^)^, nutrition education (dietitian access, cooking demonstration)^(^ ^47^ ^)^ and/or referral to social and community services (e.g. Meals on Wheels, community meal programme)^(^ ^27^ ^,^ ^29^ ^,^ ^40^ ^,^ ^41^ ^,^ ^43^ ^,^ ^44^ ^)^. Hamirudin *et al*. concluded that timely nutrition screening followed by intervention improves the nutritional status of these older adults. As a follow-up to this work, a pilot study was conducted by Hamirudin *et al*.^(^ ^48^ ^)^ in sixty-eight patients to determine if a model of home-based dietetic care improved dietary intake and weight status in a specific group of older adults (>65 years of age) following hospitalization. Individualized diet advice was provided by a dietitian as the primary intervention. Improved nutritional status was demonstrated by increases in body weight (*P*=0·048), as well as mean MNA scores (21·9 (sd 3·5) *v*. 25·2 (sd 3·1); *P*<0·001). Specifically, the use of oral nutrition supplements and milk was associated with increased overall energy intake pre- and post-intervention. Recent data also suggest oral nutrition supplementation in community-dwelling older individuals at nutrition risk may be a simple, cost-effective nutrition intervention^(^ ^49^ ^)^.

Our data provide a unique opportunity to gain insights into the nutritional concerns of an ethnically diverse group of older adults residing within an urban community. We employed methods that posed minimal participant burden and gathered data on a large number of individuals in a relatively short period of time (~4 months). Despite these methodological strengths, the study is not without limitations. First, individuals who refused to participate in our survey may be inherently different. By design, we know very little about those who refused or the ones who could not be reached by phone. Therefore, we cannot determine the degree to which non-response is biasing the results or the true generalizability of our findings. We chose a very conservative method to calculate overall response rate, basing our estimates on the sample of 64 445 potential telephone numbers. Using other less conservative methods (e.g. removing non-working numbers, only including direct contact to screener, cooperation with screener and those meeting all eligibility in the denominator) would reflect significantly higher response rates. Second, we did not gather data on depression or social support, two potentially important confounders of nutrition risk^(^ ^28^ ^–^ ^30^ ^)^ that would have added substantial time to the length to the interview. Although we did find that ‘eating alone’ was a major predictor of nutrition risk and this may be a proxy for social isolation, a recent systematic review found these two factors were not associated with documented protein–energy malnutrition in older adults^(^ ^50^ ^)^. Third, we used the abPG-SGA tool to assess the nutrition risk. This screening tool is conventionally used in the oncology setting and like other screening tools, does rely on self-reported data which then merits corroboration for further nutrition assessment. Fourth, based on the number of available covariates, the likelihood of residual confounding cannot be ruled out. Although there were 210 events (i.e. high-risk individuals), our logistic regression model was limited to fourteen predictors following the statistical methodologies of Peduzzi *et al*.^(^ ^51^ ^)^. Finally, our cross-sectional study design prohibits the establishment of cause-and-effect relationships.

## Conclusions

The present study documents that 26 % of older adults with overweight and obesity residing in an urban, community environment report signs and symptoms consistent with high nutrition risk. Additionally, Black respondents reflect the greatest proportion of those with high risk scores, yet Hispanic participants display the most concerning nutrition risk profiles. The study contributes to a growing body of research that supports the elevated nutrition risk among independent-living older adults, regardless of BMI, and offers unique findings among minority participants. It is clear that our current identification and intervention strategies are lacking, since the relative majority of research endeavours related to nutrition risk and malnutrition have focused on the inpatient hospital environment. Future studies will require comprehensive and in-depth assessment methods and, more importantly, targeted well-designed interventions to promote healthy ageing throughout our communities.
